# Markers of intestinal barrier damage in patients with chronic insomnia disorder

**DOI:** 10.3389/fpsyt.2024.1373462

**Published:** 2024-03-28

**Authors:** Yixian Cai, Di Gong, Ting Xiang, Xiaotao Zhang, Jiyang Pan

**Affiliations:** ^1^ Department of Psychiatry, Sleep Medicine Centre, The First Affiliated Hospital of Jinan University, Guangzhou, China; ^2^ Shenzhen Eye Hospital, Jinan University, Shenzhen, China; ^3^ Department of Sleep Disorders, Affiliated Wuhan Mental Health Center, Tongji Medical College of Huazhong University of Science and Technology, Wuhan, China

**Keywords:** chronic insomnia disorder, intestinal barrier, diamine oxidase (DAO), D-lactic acid (D-LA), intestinal fatty acid binding protein (I-FABP), endothelin (ET)

## Abstract

**Objective:**

Insomnia disorder stands out as one of the prevalent clinical sleep and psychiatric disorders. Prior research has unequivocally demonstrated variations in the diversity and abundance of gut microbiota among individuals with insomnia disorder. These alterations may play a direct or indirect role in the onset and progression of insomnia disorder by compromising the integrity of the intestinal barrier. This study aims to evaluate the impairment of the intestinal barrier in individuals with insomnia disorder by scrutinizing the serum functionality of this barrier.

**Materials and methods:**

45 patients with chronic insomnia disorder and 30 matched healthy volunteers were meticulously selected based on inclusion criteria. ELISA technology was employed to measure serum levels of diamine oxidase (DAO), D-lactic acid (D-LA), intestinal fatty acid binding protein (I-FABP), and endothelin (ET). Spearman correlation analysis was used to explore the relationship between intestinal mucosal markers and clinical characteristics. Data were analyzed using SPSS 26.0.

**Results:**

Compared to the healthy control group, the insomnia disorder group exhibited significantly elevated scores on subjective mood and sleep scales (GAD-7, PHQ-9, HAMA, HAMD, PSQI, and ISI) (P < 0.05). Overnight PSG indicated a notable increase in bed time, total wake time, sleep onset latency, and wake after sleep onset in individuals with insomnia disorder. Additionally, there was a decrease in sleep efficiency and alterations in sleep structure (increased proportion of N1 and N3 stages, prolonged N1 stage) (P < 0.05). The chronic insomnia disorder group displayed significantly reduced concentrations of serum DAO, D-LA, I-FABP, and ET (P < 0.05). Furthermore, significant positive correlations were identified between intestinal epithelial barrier markers and sleep efficiency, while negative correlations were found with wake after sleep onset, total wake time, PSQI, HAMA, and HAMD. Additionally, D-LA levels were significantly positively correlated with ET concentrations.

**Conclusion:**

Individuals with chronic insomnia disorder manifest disruptions in sleep structure, heightened susceptibility to anxiety and depressive moods, and impaired intestinal barrier function. These findings suggest that the occurrence and development of insomnia disorder may be linked to the impairment of the intestinal barrier.

## 1 Introduction

Insomnia disorder stands out as one of the most prevalent clinical sleep and mental health challenges, affecting approximately 30% of individual (1). Consistently identified risk factors include being female, advanced age, and experiencing concomitant psychiatric disorders, and concomitant obstructive sleep apnea (2, 3). Beyond its impact on patients’ social and cognitive functions, insomnia disorder escalates the risk of developing significant physical ailments, such as depression, anxiety disorders, cardiovascular diseases, immune disorders, dementia, and cancer (4, 5). This not only places a substantial economic and healthcare burden on patients, their families, and society but also underscores its status as an urgent public health concern in need of resolution (6, 7).

In recent years, a plethora of research has confirmed the involvement of gut microbiota via the microbiota-gut-brain axis in various systemic diseases, including insomnia disorders, schizophrenia, and depressive disorders, among other mental illnesses (8, 9). Gut microbiota not only influence the host’s digestion, immune, and metabolic functions but also regulate sleep and mental states through the microbiota-gut-brain axis, wherein the dynamic regulation of the intestinal mucosal barrier plays a crucial role (10). It has been established that the intestinal mucosal barrier is compromised in several mental disorders. Previous studies have substantiated changes in the diversity and abundance of gut microbiota among individuals with insomnia disorder. The potential impact of an imbalanced gut microbiota on brain function suggests a direct or indirect involvement in the onset and progression of insomnia disorder (8). This has been confirmed in sleep deprived mice (11). Nevertheless, the precise mechanisms through which gut microbiota participates in the pathogenesis of insomnia disorder remain not fully elucidated, with intestinal barrier function likely playing a pivotal role in these processes.

The integrity of the intestinal barrier is paramount for maintaining intestinal homeostasis (12–14). When the function of the intestinal barrier is compromised, the mucosal barrier may undergo “leakage,” permitting the entry of pathogens and toxins into the body. This, in turn, triggers inflammatory reactions and, in severe cases, can result in multi-organ failure (15). Diamine oxidase (DAO), also known as histamine oxidase, is widely distributed in various tissues, with a notable presence in the mucosa of the small intestine. DAO is found at low levels in the blood, and its activity serves as an indicator of intestinal mucosal maturity and integrity, making it a reliable measure for monitoring intestinal mucosal function (16, 17). D-Lactic acid (D-LA) is exclusively produced through bacterial metabolism in the intestines and can provide insights into changes in intestinal ischemia and permeability (18). Endotoxin (ET), originating from gram-negative bacteria, serves as a marker for assessing the disruption of the intestinal barrier function. When the intestinal barrier function is impaired, a substantial amount of ET produced by gram-negative bacteria in the intestines enters the bloodstream, leading to an imbalance in blood ET levels (19, 20). Intestinal fatty acid binding protein (I-FABP) is a protein found in mature small intestine epithelial cells, acting as a marker for intestinal epithelial cell integrity. Under normal circumstances, the concentration of I-FABP in tissues is relatively high, while its concentration in serum remains low. However, when the intestinal mucosa is damaged, I-FABP is released into the circulation, resulting in an elevation of its serum concentration (21). These indicators collectively offer a quantitative assessment of intestinal barrier function.

Therefore, we hypothesize differences in intestinal barrier biomarkers among individuals with insomnia disorder. To examine this hypothesis, we assessed disparities in intestinal barrier markers between patients with insomnia disorder and a healthy control group, exploring their associations with clinical characteristics.

## 2 Materials and methods

### 2.1 Subjects

This study is a case-control research adhering strictly to inclusion and exclusion criteria, enrolling participants in both the insomnia disorder group and the healthy control group. All subjects underwent clinical assessments and diagnoses conducted independently by two attending psychiatrists from the First Affiliated Hospital of Jinan University. Inclusion in the study required a unanimous diagnostic opinion from both researchers regarding the participants. Under the guidance of medical professionals, all subjects completed general questionnaires, clinical interviews, screening with clinical scales, and polysomnography (PSG). Only those meeting the diagnostic criteria were included in the study.

All participants were of Han Chinese ethnicity, aged between 18 and 65, and met the criteria for chronic insomnia disorder according to ICSD-3. They also had a Pittsburgh Sleep Quality Index (PSQI) score of ≥8. Exclusion criteria included a history of other sleep disorders, mental illnesses, or severe physical illnesses. Participants had no specific dietary habits (such as vegetarianism or traditional ethnic diets) and had not undergone surgery, taken antibiotics, or used probiotics in the past two months.

Matched healthy volunteers were spouses or socially recruited individuals unrelated to the patients, aged 18 to 65, with a PSQI score of ≤7 and no history of mental or severe physical illnesses.

Ethics committee approval for the study was granted by the Medical Ethics Committee of the First Affiliated Hospital of Jinan University (IRB of First Affiliated Hospital of Jinan University, No. KY-2022-167). All participants provided written informed consent before participating in the study, and researchers adhered to the Declaration of Helsinki Principles.

### 2.2 PSG

All participants underwent two nights of PSG to exclude other sleep disorders and first-night effects. Participants followed their usual sleep habits for bedtime and wake-up time, and provided samples the morning after waking up. PSG results were interpreted according to the standards outlined in the American Academy of Sleep Medicine (AASM) Manual for the Scoring of Sleep and Associated Events, Version 2.6. Objective PSG parameters included total bed time (TBT), total sleep time (TST), sleep onset latency (SOL), sleep efficiency (SE), wakefulness after sleep onset time (WASO), rapid eye movement (REM), as well as time and proportion of each sleep stage (N1, N2, N3, REM). The data from the second night of PSG were used as parameters in this study.

### 2.3 Blood sampling and ELISA testing

Blood samples were collected from the antecubital vein on an empty stomach within one hour after waking up on the morning following the second night of PSG monitoring from all participants. A total of 5 ml was collected to measure the serum concentrations of DAO, D-LA, I-FABP, and ET. After allowing the samples to rest for 30 minutes, they were centrifuged at 3000 rpm for 10 minutes at 4°C to separate the serum. The serum was then aliquoted into sterile Eppendorf tubes and immediately stored at -80°C for future use.

Human serum levels of DAO, D-LA, I-FABP, and ET were detected using enzyme-linked immunosorbent assay (ELISA) kits (JIANGSU BOSHEN BIOTECHNOLOGY Co., Ltd、JSBOSSEN). The procedure involved steps such as sample addition, enzyme addition, incubation, washing, and color development. Standards, control sera, and samples are run in duplicate in each assay. Finally, the optical density (OD) of each well was measured at a wavelength of 450 nm, and the concentrations were calculated accordingly.

### 2.4 Statistical analysis

The data were analyzed using SPSS 26.0 for descriptive statistics and inferential analysis. Normality tests were conducted for all data, and for normally distributed metric data, mean (standard deviation) was used. Group comparisons were performed using the t-test. For non-normally distributed metric data, median (P25, P75) was used, and group comparisons were conducted using the Mann-Whitney U test. Categorical data were analyzed using the χ2 test, and correlation analysis was performed using Spearman correlation analysis. All tests were two-tailed, and statistical significance was set at p < 0.05.

## 3 Results

A total of 73 individuals with insomnia disorder were recruited for this study. Among them, 28 participants were excluded due to recent use of sleep aids, diagnosis of comorbid obstructive sleep apnea syndrome, periodic limb movement disorder, or other mental illnesses. The final insomnia disorder group (ID group) comprised 45 patients, and blood samples were collected from all of them. Additionally, 42 healthy volunteers were recruited. Twelve participants were excluded due to non-cooperation, diagnosis of obstructive sleep apnea syndrome, or periodic limb movement disorder identified through polysomnography. The healthy control group (HC group) ultimately included 30 participants, and blood samples were collected from all of them. In the HC group, the gender distribution was 10 males and 20 females, with a median age of 32 years and an average Body Mass Index (BMI) of (22.19 ± 2.57) kg/m2. In the ID group, the gender distribution was 18 males and 27 females, with a median age of 42 years and an average BMI of (21.99 ± 2.90) kg/m2. There were no statistically significant differences between the HC and ID groups in terms of gender, age, and BMI (P>0.05) (**Table 1**).

**Table 1:** Clinical baseline data of ID and HC groups.

	Insomnia Disorder Group (ID) N=45	Healthy control group (HC) N=30	*P.*overall
Baseline information			
Females, n(%)	27 (60.00%)	20 (66.67%)	0.559
Age (years)	42.00 (27.5,53.0)	32.00 (26.0,48.3)	0.258
BMI(kg/m^2^)	21.99 ± 2.90	22.19 ± 2.57	0.754
PHQ-9	6.00 (3.0,7.0)	1.00 (0.0,2.0)	<0.001^***^
GAD-7	4.00 (1.0,7.5)	1.00 (0.0,2.3)	0.001^**^
PSQI	13.00 (10.0,16.0)	4.00 (2.0,5.0)	<0.001^***^
ESS	6.00 (3.0,10.0)	5.00 (3.0,7.3)	0.412
ISI	16.00 (10.5,19.5)	2.50 (1.0,6.0)	<0.001^***^
HAMA	5.00 (3.0,8.5)	0.00 (0.0,1.0)	<0.001^***^
HAMD	6.00 (3.0,7.5)	0.000 (0.0,1.0)	<0.001^***^
PSG			
TBT (min)	515.46 ± 56.77	452.70 ± 57.20	<0.001^***^
TST(min)	384.52 ± 78.39	386.75 ± 62.22	0.896
WASO (min)	85.50 (55.3,144.0)	35.00 (22.3,64.0)	<0.001^***^
SE(%)	77.90 (0.7,0.8)	86.90 (0.8,0.9)	<0.001^***^
SOL(min)	11.00 (7.5,32.8)	7.50 (4.3,25.0)	0.037^*^
N1(min)	46.50 (35.3,65.0)	37.75 (23.3,52.6)	0.013^*^
N1(%)	12.00 (9.0,18.2)	8.45 (5.4,14.0)	0.005^**^
N2(min)	189.73 ± 57.13	190.88 ± 47.79	0.928
N2(%)	49.50 (42.5,55.6)	48.60 (43.1,56.2)	0.974
N3(min)	59.43 ± 29.12	76.92 ± 43.43	0.040^*^
N3(%)	14.90 (9.4,21.2)	18.800 (11.5,27.7)	0.104
REM(min)	76.96 ± 29.95	79.62 ± 29.15	0.704
REM(%)	19.700 (15.6,23.6)	19.600 (15.3,25.6)	0.520
TWT(min)	114.500 (74.3,156.5)	54.00 (34.9,72.6)	<0.001^***^

*P<0.05, ** P <0.01, *** P <0.001.

In subjective mood assessments, individuals with insomnia disorder exhibited significantly higher scores for anxiety and depression (P<0.001). Regarding subjective sleep quality and insomnia severity, the insomnia disorder group had markedly higher scores in both the Pittsburgh Sleep Quality Index (PSQI) and the Insomnia Severity Index (ISI) compared to the healthy control group (P<0.001). Furthermore, in terms of objective sleep measures, significant differences were observed between the two groups at multiple levels. The insomnia disorder group showed a substantial increase in total time in bed, total wake time, sleep onset latency, and wake after sleep onset. Sleep efficiency was reduced, and alterations in sleep structure were evident, including an increase in N1 stage time and proportion and a decrease in N3 stage time (P<0.05). These clinical phenotypes align with findings from previous research.

In subjective mood assessments, individuals with insomnia disorder exhibited significantly higher scores for anxiety and depression (P<0.001). Regarding subjective sleep quality and insomnia severity, the insomnia disorder group had markedly higher scores in both the Pittsburgh Sleep Quality Index (PSQI) and the Insomnia Severity Index (ISI) compared to the healthy control group (P<0.001). Furthermore, in terms of objective sleep measures, significant differences were observed between the two groups at multiple levels. The insomnia disorder group showed a substantial increase in total time in bed, total wake time, sleep onset latency, and wake after sleep onset. Sleep efficiency was reduced, and alterations in sleep structure were evident, including an increase in N1 stage time and proportion and a decrease in N3 stage time (*P*<0.05). These clinical phenotypes align with findings from previous research.

**Figure 1:**
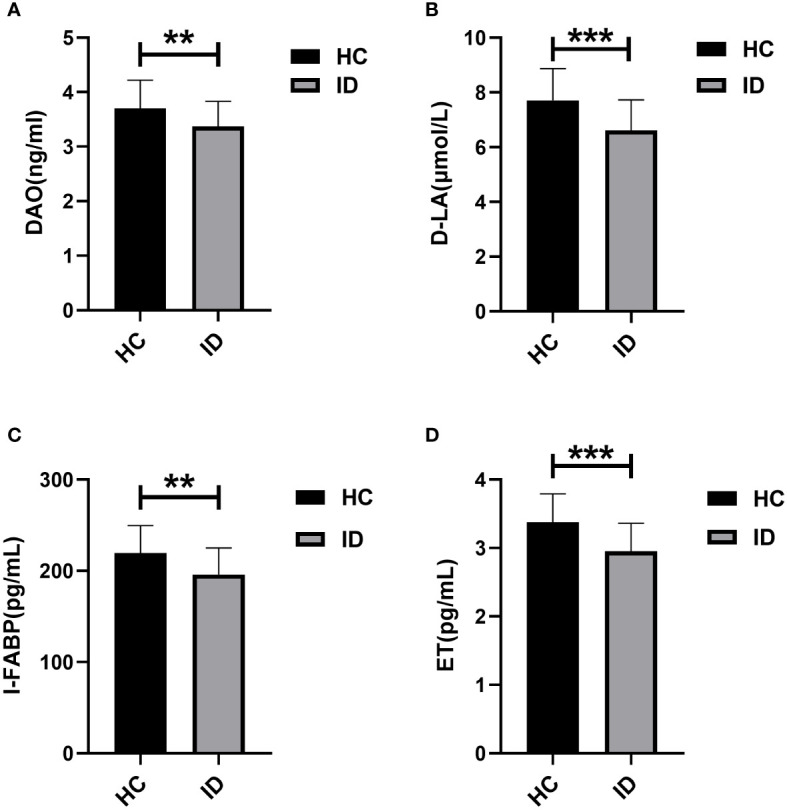
Comparison of serum levels of DAO, D-LA, I-FABP, and ET between ID and HC. ** P <0.01, *** P <0.001.

Levels of serum intestinal mucosal damage markers, including diamine oxidase (DAO), D-lactic acid (D-LA), intestinal fatty acid binding protein (I-FABP), and serum endotoxin (ET), in the ID group and HC group are presented in **Table 2**. Significant differences were observed in the levels of these markers between individuals with insomnia disorder and the healthy control group (DAO (3.37 ± 0.46) vs (3.70 ± 0.52) ng/mL, D-LA (6.61 ± 1.12) vs (7.71 ± 1.16) μmol/L, I-FABP (195.87 ± 29.19) vs (219.29 ± 30.42) pg/mL) and ET (2.95 ± 0.41) vs (3.38 ± 0.41) pg/mL) (*P* < 0.001), with notably lower levels in the insomnia disorder group compared to the healthy control group (**Figure 1**).

**Table 2:** Comparison of serum DAO, D-LA, I-FABP, and ET levels between ID and HC.

	ID (N=45)	HC (N=30)	*p*
DAO (ng/mL) D-LA (μmol/L) I-FABP (pg/mL) ET (pg/mL)	3.37 ± 0.46 6.61 ± 1.12 195.87 ± 29.19 2.95 ± 0.41	3.70 ± 0.52 7.71 ± 1.16 219.29 ± 30.42 3.38 ± 0.41	0.005** <0.001*** 0.001** <0.001***

* P <0.05, ** P <0.01, *** P <0.001.

**Figure 2:**
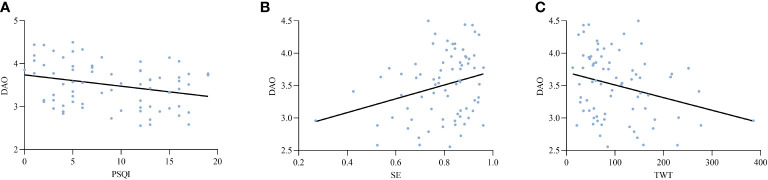
Comparison between serum levels of DAO and PSQI **(A)**, SE **(B)**, TWT **(C)**.

**Figure 3:**
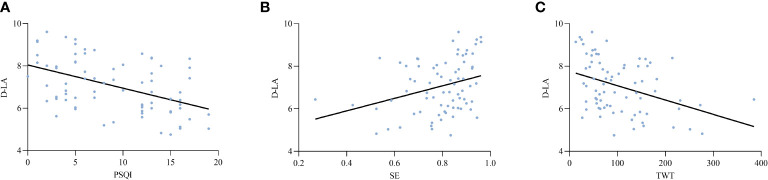
Comparison between serum levels of D-LA and PSQI **(A)**, SE **(B)**, TWT **(C)**.

**Figure 4:**
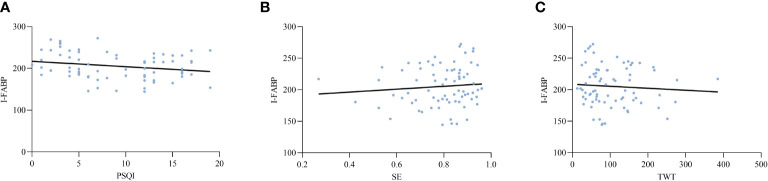
Comparison between serum levels of I-FABP and PSQI **(A)**, SE **(B)**, TWT **(C)**.

**Figure 5:**
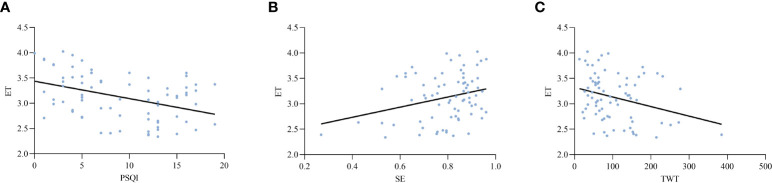
Comparison between serum levels of ET and PSQI **(A)**, SE **(B)**, TWT **(C)**.

Based on the above findings, an analysis was conducted to explore the potential relationships between intestinal barrier markers and sleep indices. The results revealed a significant positive correlation between markers of intestinal epithelial barrier function and sleep efficiency. Additionally, these markers showed a negative correlation with wake after sleep onset (WASO), total wake time (TWT), Pittsburgh Sleep Quality Index (PSQI), Hamilton Anxiety Rating Scale (HAMA), and Hamilton Depression Rating Scale (HAMD). Furthermore, there was a significant positive correlation between D-lactic acid (D-LA) levels and endotoxin (ET) concentrations (**Table 3**). **Figures 2**–**4** depict the correlations between intestinal barrier markers and PSQI, sleep efficiency (SE), and TWT among the 75 subjects (**Figure 2A**, *R ^2^* = -0.279; **Figure 2B**, *R ^2^ =* 0.248; **Figure 2C**, *R^2^* =-0.260; **Figure 3A**, *R ^2^* = -0.449; **Figure 3B**, *R* ^2^ = 0.338; **Figure 3C**, R^2^=-0.3970; **Figure 4A**, *R* ^2^ = -0.223; **Figure 4B**, *R* ^2^ = 0.134; **Figure 4C**, *R^2^* =-0.103; **Figure 5A**, *R* ^2^= -0.379; **Figure 5B**, *R* ^2^ = 0.247; **Figure 5C**, R^2^=-0.271).

**Table 3:** The potential associations between variables and D-LA,DAO, I-FABP and ET.

	DAO		D-LA		I-FABP		ET	
	*r*	*p*	*r*	*p*	*r*	*p*	*r*	*p*
PSQI	*-0.279*	0.025*	*-0.449*	<0.001^***^	-0.223	0.055	*-0.379*	0.001^***^
HAMA	*-0.338*	0.049*	*-0.426*	<0.001^***^	*-0.264*	0.022^*^	*-0.281*	0.015^*^
HAMD	*-0.286*	0.013*	*-0.461*	<0.001^***^	-0.220	0.057	*-0.305*	0.008^**^
SE	*0.248*	0.043*	*0.338*	0.003^**^	0.134	0.254	*0.247*	0.033^*^
WASO	-0.172	0.140	*-0.344*	0.003^**^	-0.132	0.258	*0.274*	0.017^*^
TWT	*-0.260*	0.024*	*-0.397*	<0.001^***^	-0.103	0.380	*0.271*	0.019^*^
D-LA	0.099	0.400	–	–	–	–	–	–
I-FABP	0.170	0.146	0.017	0.886	–	–	–	–
ET	0.029	0.807	*0.239*	*0.039*	0.158	0.176	–	–

** P <0.01, *** P <0.001.

## 4 Discussions

Insomnia disorder, being one of the most common sleep disorders and mental illnesses in clinical practice, imposes numerous adverse effects on individuals, families, and society. The clinical challenges and the increasing trend in its prevalence make in-depth research into the mechanisms, diagnosis, and treatment of insomnia disorder imperative (22). Current studies suggest that alterations in gut microbiota occur in individuals with insomnia disorder, indicating the potential involvement of the microbiota-gut-brain axis in the development of insomnia disorder. However, the specific mechanisms of this involvement remain unclear. Previous research has also confirmed abnormalities in intestinal mucosal damage markers in severe mental illnesses such as schizophrenia and sleep disorders like obstructive sleep apnea syndrome, suggesting damage to the intestinal mucosa (23, 24). This study aims to clarify whether there are abnormalities in the intestinal barrier of individuals with insomnia disorder by examining serum markers of intestinal mucosal damage.

This study utilized ELISA technology to detect serum markers of intestinal barrier damage in individuals with insomnia disorder and the healthy control group. The results indicated that serum levels of diamine oxidase (DAO), D-lactic acid (D-LA), intestinal fatty acid binding protein (I-FABP), and endotoxin (ET) in individuals with insomnia disorder were significantly lower than those in the healthy control group (P<0.001). This suggests potential impairment in the function of the intestinal barrier or possible atrophy of the intestinal mucosa in individuals with insomnia disorder. Additionally, significant correlations were found between DAO, D-LA, I-FABP, ET, and sleep efficiency, total wake time, and sleep quality (PSQI). The poorer the quality of sleep, the longer the total sleep wakefulness, the lower the sleep efficiency, and the more severely the serum gut barrier is compromised in patients with insomnia disorders.

The physiological functions of diamine oxidase (DAO) primarily include participation in the regulation of inflammation, allergic reactions, and ischemic processes (25). Intestinal barrier integrity is typically assessed through intestinal morphology, serum DAO activity, and D-lactic acid (D-LA) activity. DAO activity and changes in D-LA levels serve as biomarkers for intestinal permeability, and alterations in their levels indicate changes in intestinal mucosal maturity or integrity (26, 27). Previous studies have shown a positive correlation between DAO activity and intestinal mucosal permeability. However, in this study, DAO was significantly reduced compared to healthy individuals, suggesting a decrease in intestinal mucosal maturity or permeability, possibly even mucosal atrophy. A decrease in DAO activity can lead to an increase in plasma histamine concentration, resulting in symptoms of “histamine intolerance” such as diarrhea, hypotension, and headaches (28). Previous research has indicated reduced DAO activity in individuals with anorexia nervosa (27). Additionally, studies have found decreased DAO in cancer patients, with histological examinations suggesting a reduction in villus height and surface area. There was a significant correlation between the percentage decrease in DAO activity and the severity of diarrhea (29, 30).

Intestinal fatty acid binding protein (I-FABP) is expressed in the tips of intestinal villi. Once the integrity of the cell membrane is compromised, mature intestinal cells release this cytoplasmic protein. Therefore, I-FABP reflects the degree of intestinal damage (31). I-FABP also participates in fatty acid metabolism by assisting intestinal cells in absorbing fatty acids from the intestinal lumen and bloodstream. Previous studies have suggested its potential role in lipid metabolism, regulation of intestinal structure, and nutrient absorption, thereby influencing overall energy metabolism (32, 33). Individuals with insomnia disorder often report close associations with gastrointestinal symptoms such as indigestion and diarrhea. The decrease in I-FABP may be attributed to poor absorption of fats by intestinal cells (34).

Currently, there is a lack of relevant research related to the relationship between several intestinal mucosal barrier markers and clinical features in patients with insomnia disorder. However, previous studies in severe mental illnesses like schizophrenia have shown an elevation in markers of intestinal mucosal damage, indicating intestinal mucosal damage and increased permeability. On the other hand, consistent with the findings in this study, individuals with anorexia nervosa exhibited decreased intestinal mucosal permeability. Regarding the results of this study, several possibilities may contribute: Chronic Course of Insomnia Disorder: Insomnia disorder is a chronic condition, and the decreased levels of serum markers (DAO, D-LA, I-FABP, ET) observed in individuals with insomnia disorder may lead to impaired intestinal barrier function and reduced intestinal permeability, reflecting a compensatory phase in the body. Alterations in Gut Microbiota: The results indicate dysregulation in the gut microbiota of individuals with insomnia disorder. The decrease in serum markers suggests damage to the intestinal barrier, emphasizing a close association between gut microbiota and intestinal barrier in insomnia disorder patients. These findings highlight the need for further research in the diagnosis and treatment of individuals with insomnia disorder, potentially opening new directions for understanding and addressing the condition.

In this study, the evaluation of intestinal barrier function was conducted by detecting the concentrations or activities of DAO, ET, I-FABP, and D-LA in serum. While previous research has consistently shown that these markers can indicate intestinal barrier function, they do not provide direct feedback on the specific location and extent of damage within the intestinal mucosa. In future studies, it is recommended to use pathological tissue sections of the intestinal mucosa from patients for a more accurate depiction of the intestinal mucosal condition.

Furthermore, it would be beneficial to validate whether these markers influence the prognosis of individuals with insomnia disorder and other biochemical indicators. As research on gut microbiota has advanced in recent years, revealing age-related differences in gut microbiota, this study, limited by the number of subjects, did not compare gut microbiota in individuals with insomnia disorder across different age groups. Future research should consider increasing the sample size and conducting age-stratified studies to explore potential variations in gut microbiota among different age stages of individuals with insomnia disorder.

## 5 Conclusion

Patients with chronic insomnia disorder have disturbed sleep structure, are more likely to have anxiety and depression, and are accompanied by impaired intestinal barrier function, suggesting that insomnia disorder may be involved in the development of insomnia disorder by damaging the intestinal barrier.

## Data availability statement

The raw data supporting the conclusions of this article will be made available by the authors, without undue reservation. Requests to access these datasets should be directed to YC, YixianCai2020@163.com.

## Ethics statement

The studies involving humans were approved by the Medical Ethics Committee of the First Affiliated Hospital of Jinan University (IRB of First Affiliated Hospital of Jinan University, No. KY-2022-167). The studies were conducted in accordance with the local legislation and institutional requirements. The participants provided their written informed consent to participate in this study. Written informed consent was obtained from the individual(s) for the publication of any potentially identifiable images or data included in this article.

## Author contributions

YC: Project administration, Methodology, Investigation, Writing – review & editing, Writing – original draft, Data curation, Conceptualization. DG: Writing – review & editing, Writing – original draft, Validation, Software, Methodology, Data curation. TX: Writing – review & editing, Writing – original draft, Validation, Supervision, Methodology, Investigation, Data curation. XZ: Writing – review & editing, Writing – original draft, Validation, Methodology, Investigation, Formal analysis, Data curation. JP: Writing – review & editing, Writing – original draft, Validation, Supervision, Resources, Project administration, Funding acquisition, Conceptualization.
